# Sympathetic ophthalmia with sensorineural deafness - report of a case

**DOI:** 10.1186/1869-5760-3-65

**Published:** 2013-11-13

**Authors:** Ramesh Venkatesh, Manisha Agarwal, Vidya Janaki Ramesh, Shalini Singh, Meha Kantha, Jyotirmay Biswas

**Affiliations:** 1 Eye Department, Dr Shroff Charity Eye Hospital, 5027, Kedarnath Road, Daryaganj, New Delhi 110002, India; 2 ENT Department, Dr Shroff Charity Eye Hospital, 5027, Kedarnath Road, Daryaganj, New Delhi 110002, India; 3 Uvea and Ocular Pathology Department, Sankara Nethralaya, 18, College Road, Numgambakkam, Chennai 600006, India

**Keywords:** Sympathetic ophthalmia, Deafness, Corticosteroids, Psychosis

## Abstract

**Background:**

The aim of this study is to report a case of sympathetic ophthalmia with sensorineural hearing loss following penetrating trauma. This is an interventional case report. A 23-year-old male presented with bilateral, sudden, profound visual and hearing loss, disorientation, and dizziness. He had a past history of penetrating trauma with an iron rod in the right eye for which he underwent scleral tear repair, vitreo-retinal surgery with intraocular foreign body removal and silicon oil injection. His best corrected visual acuity in the right eye was counting fingers close to the face and was perception of light in the left eye. Clinical evaluation with slit biomicroscopy, indirect ophthalmoscopy, ultrasonography, and pure tone audiometry was suggestive of sympathetic ophthalmia with sensorineural hearing loss. Treatment was started with intravenous methyl prednisolone, oral corticosteroids, and immunosuppressants.

**Findings:**

Following treatment, signs of panuveitis showed resolution and improvement in visual, hearing, and neurological symptoms.

**Conclusions:**

Sympathetic ophthalmia associated with sensorineural deafness and neurological symptoms is a rare clinical syndrome. Prompt diagnosis and treatment with systemic corticosteroids and immunosuppressant medication may result in clinical improvement.

## Findings

### Introduction

Sympathetic ophthalmia (SO) is a rare bilateral granulomatous panuveitis which occurs after surgical or nonsurgical trauma. We report a case of sympathetic ophthalmia associated with sensorineural deafness following penetrating trauma with an intraocular foreign body to one eye. Visual and hearing loss showed improvement after treatment with oral corticosteroids and immunosuppressant drugs.

### Case description

A 23-year-old male patient presented with sudden diminution of vision in both eyes and bilateral hearing loss for the last 15 days. He had a history of penetrating injury to the right eye with an iron rod. He had a history of scleral tear repair followed by a vitreo-retinal surgery including metallic intraocular foreign body removal and silicone oil injection 3 months prior to presentation. Postoperatively, the vision had improved to 3/60, 6/24 reduced Snellen's visual acuity (RS) with a well-attached retina and silicone oil *in situ* in the right eye.

**Figure 1:**
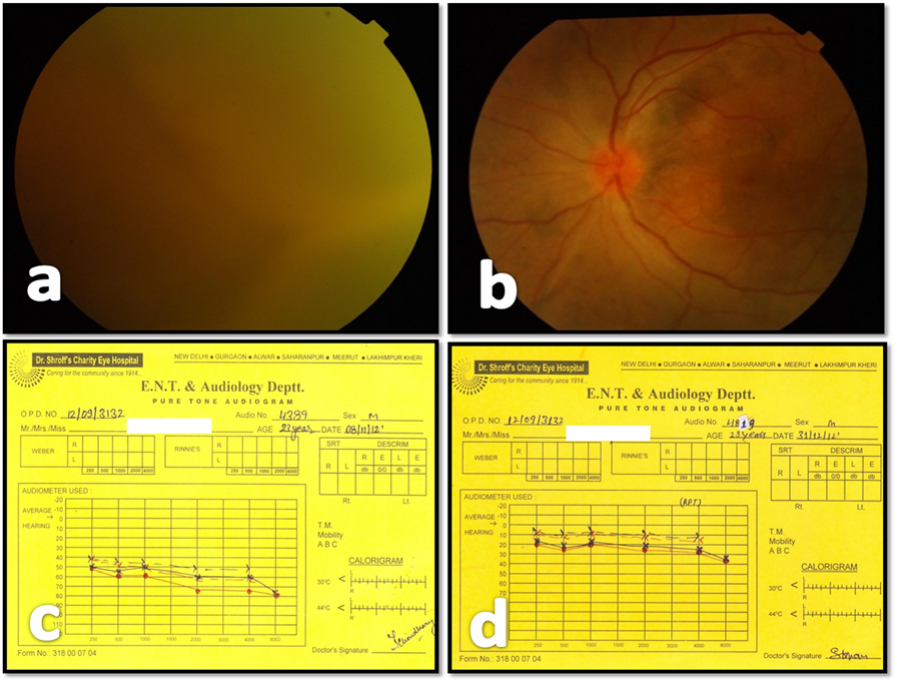
**Pre- and posttreatment color fundus and pure tone audiometry findings. (a)** Color fundus photo of the left eye (LE) with dense vitritis with underlying exudative retinal detachment. **(b)** Color fundus photo LE. Posttreatment with corticosteroids and immunosuppressants, vitritis has resolved with the presence of inferior retinal detachment. **(c**, **d)** Pre- and post-treatment audiograms respectively showing improvement in hearing threshold from 60 to 20 dB following treatment with steroids and immunosuppressants.

On systemic evaluation by our general physician, the patient was not obeying verbal commands, was restless and anxious, and disoriented to time, place, and person. The best corrected visual acuity (BCVA) in the right eye was counting fingers close to the face and was perception of light in the left eye. Slit lamp examination of the right eye showed moderate anterior uveitis with anterior chamber cells 2+ and flare 2+ with aphakia and inferior peripheral iridectomy. Fundus examination of the right eye showed a hyperemic optic disc with a large retinal break surrounded by laser chorio-retinal atrophy in the supertemporal quadrant and silicone oil *in situ*. The left eye showed pigmented keratic precipitates, anterior chamber cells 3+, flare 3+, and retrolental cells in the anterior vitreous. The left eye showed dense media haze due to grade 4 vitritis [1] with retinal detachment which could just be discerned (Figure 1a). Intraocular pressure in both eyes was 9 mm Hg (Goldmann Applanation tonometer). Ultrasound B-scan showed shifting fluid confirming the presence of an exudative retinal detachment with increased choroidal thickness measuring 2.3 mm in the left eye. Fundus fluorescein angiography was not done on initial presentation. Pure tone audiometry showed a sensorineural deafness for both lower and higher frequencies with a hearing threshold of 60 dB (Figure 1c).

Systemic and neurological examinations were performed by our physician. Investigations such as Mantoux test 3 × 5 mm of induration, ESR = 15 mm/h, normal chest X-ray, negative urine and blood cultures, and negative HIV and VDRL were done to rule out other systemic infections. MRI of the brain was normal. CSF examination was not done as the patient was not willing to undergo the procedure.

On examination, there was evidence of minimal inflammation in the right eye and severe inflammation with exudative detachment in the left eye. He was diagnosed with sympathetic ophthalmia. Topical prednisolone acetate (1%) instilled every hour and atropine (1%) three times a day were started for the left eye. Topical prednisolone acetate (1%) every 4 h was added for the right eye. Intravenous methylprednisolone (1.5 mg/kg weight) for three consecutive days followed by oral corticosteroid (1.5 mg/kg weight) in a tapering dose was started. Oral azathioprine (1.5 mg/kg weight) was added later.

Follow-up at 2 weeks showed improvement in the patient's general condition; he was following verbal commands and had a good orientation to time, place, and person. On ocular examination, BCVA in the right eye was counting fingers close to the face and was 6/36, 6/9 RS in the left eye. Anterior segment evaluation showed mild anterior uveitis. Fundus examination showed reduction in vitritis to grade 2 [1] with the presence of exudative retinal detachment in the left eye. Intraocular pressure (IOP) was 12 mm Hg in both eyes.

At 9 months, the BCVA in the right eye was counting fingers close to the face and 6/36, 6/9 RS in the left eye. The anterior segment was quiet. IOP was 12 mm Hg in both eyes. Fundus examination showed a shallow inferior exudative retinal detachment in sitting position (Figure 1b).

There was evidence of extensive areas of hyperfluorescence in the early phase with increase in intensity and then fading out without changing size and shape in the late phase of the fluorescein angiogram, suggestive of transmission window defects due to the atrophy of the retinal pigment epithelium. A pure tone audiometry was done which showed improvement in the hearing threshold to 20 dB (Figure 1d).

**Figure 2:**
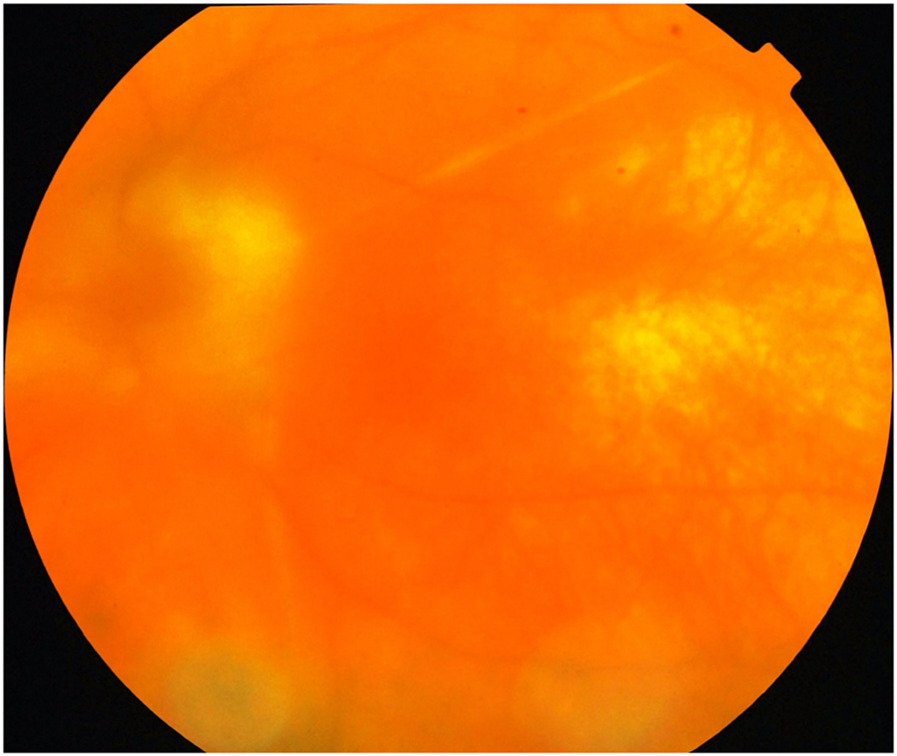
**Color fundus findings on final follow-up.** Color fundus image of the left eye showing sunset glow fundus and complete resolution of the subretinal fluid. Gliosis surrounding the optic disc is noted.

On last follow-up at 15 months, the BCVA in the right eye was counting fingers close to the face and was 6/36, 6/9 RS in the left eye. The anterior segment was quiet. Fundus examination showed a sunset glow fundus with complete depigmentation of the retinal pigment epithelium and resolution of the subretinal fluid (Figure 2).

## Discussion

Sympathetic ophthalmia and Vogt Koyanagi Harada (VKH) disease cause bilateral granulomatous panuveitis. They resemble both clinically and histologically though the severity of uveitis may differ. The exciting cause of uveitis is an auto-allergy to the uveal pigment. Hearing loss is a rare association with SO and is more often found in VKH along with a clinical syndrome.

In our case, the only signs of active inflammation that we could appreciate in exciting the right eye were moderate anterior chamber cells and flare. However, the other signs of inflammation such as retrolental cells, vitritis, and increased choroidal thickness were not seen due to silicone oil *in situ*. Therefore, though bilateral, the signs of diffuse inflammation were recorded only in the left eye.

Our case of SO presented with restlessness, anxiety, and disorientation. These findings were described as ‘black patch psychosis’ by Singh et al. [2]. Black patch psychosis is said to occur in patients who have an additive influence of simultaneous loss of both visual and sensory inputs.

At 9 months follow-up, the left eye showed a persistent inferior retinal detachment. This may be explained by the following two mechanisms: firstly, it is due to the increased production of subretinal fluid secondary to a persistent subclinical active inflammation, and secondly, it is due to the decreased absorption of the subretinal fluid by the malfunctioning of the RPE pump and loss of the RPE cells due to chronic inflammation. Hence, our patient's oral corticosteroids and immunosuppressant drugs was continued. On final follow-up, there was a complete resolution of the subretinal fluid. The need for continuing oral corticosteroids and immunosuppressive agents for a longer duration is therefore justified.

Sensorineural deafness in SO has rarely been reported [3,4]. The possible cause for this dysacousis might be related to a disturbance of the pigment auditory labyrinth. The function of this pigment is unknown, but the labyrinth pigment and ocular pigment arise from the same embryonic structure of the neural crests and may be common targets of autoimmunity [5].

On review of literature, we found one case of SO with deafness reported by Comer et al. [4]. This patient was treated with oral cyclosporine and prednisolone with ultimate improvement in visual acuity; however, the hearing threshold had progressively reduced to 90 dB. Our patient differs from this case as it showed improvement in the sensorineural deafness to 20 dB.

We report this case to highlight the rare association of SO with black patch psychosis and bilateral sensorineural deafness which showed an improvement on prompt diagnosis and treatment with high-dose corticosteroids and immunosuppressant drugs.

### Consent

Written informed consent was obtained from the patient for the publication of this report and any accompanying images. Approval was granted to us by our own institute's IRB.

## Abbreviations

BCVA: Best corrected visual acuity; PL: Perception of light; RS: Reduced Snellen's visual acuity; SO: Sympathetic ophthalmia; VKH: Vogt Koyanagi Harada.

## Competing interests

The authors declared that they have no competing interests.

## Authors’ contributions

RV obtained the data and drafted the manuscript. MA was involved in drafting the manuscript and revising it critically. JB was involved in revising the manuscript. SS obtained the data. MK was responsible for the acquisition of data and intellectual content. VJR obtained and analyzed the pure tone audiogram findings of the patient. All authors read and approved the final manuscript.
